# Current Landscape and Future Directions Regarding Generative Large Language Models in Stroke Care: Scoping Review

**DOI:** 10.2196/76636

**Published:** 2025-08-07

**Authors:** XingCe Zhu, Wei Dai, Richard Evans, Xueyu Geng, Aruhan Mu, Zhiyong Liu

**Affiliations:** 1 School of Medicine and Health Management Tongji Medical College Huazhong University of Science and Technology Wuhan China; 2 Faculty of Computer Science Dalhousie University Halifax, NS Canada; 3 Department of Physiology and Pathophysiology School of Basic Medical Sciences Peking University Health Science Center Beijing China; 4 School of Ethnology and Sociology Inner Mongolia University Hohhot China

**Keywords:** large language model, stroke, generative artificial intelligence, health care, artificial intelligence, AI

## Abstract

**Background:**

Stroke has a major impact on global health, causing long-term disability and straining health care resources. Generative large language models (gLLMs) have emerged as promising tools to help address these challenges, but their applications and reported performance in stroke care require comprehensive mapping and synthesis.

**Objective:**

The aim of this scoping review was to consolidate a fragmented evidence base and examine the current landscape, shortcomings, and future directions in the design, reporting, and evaluation of gLLM-based interventions in stroke care.

**Methods:**

In this scoping review, which adhered to the PRISMA-ScR (Preferred Reporting Items for Systematic Reviews and Meta-Analyses extension for Scoping Reviews) guidelines and the Population, Concept, and Context (PCC) framework, we searched 6 major scientific databases in December 2024 for gLLM-based interventions across the stroke care pathway, mapping their key characteristics and outcomes.

**Results:**

A total of 25 studies met the predefined eligibility criteria and were included for analysis. Retrospective designs predominated (n=16, 64%). Key applications of gLLMs included clinical decision-making support (n=10, 40%), administrative assistance (n=9, 36%), direct patient interaction (n=5, 20%), and automated literature review (n=1, 4%). Implementations mainly used generative pretrained transformer models accessed through task-prompted chat interfaces. In total, 5 key challenges were identified from the included studies during the implementation of gLLM-based interventions: ensuring factual alignment, maintaining system robustness, enhancing interpretability, optimizing efficiency, and facilitating clinical adoption.

**Conclusions:**

The application of gLLMs in stroke care, while promising, remains relatively new, with most interventions reflecting early-stage or relatively simple implementations. Against this backdrop, critical gaps in research and clinical translation persist. To support the development of clinically impactful and trustworthy applications, we propose an actionable framework that prioritizes real-world evidence, mandates transparent technical reporting, broadens evaluation beyond output accuracy, strengthens validation of advanced task adaptation strategies, and investigates mechanisms for safe and effective human-gLLM interaction.

## Introduction

### Background

Stroke represents a leading cause of global morbidity and long-term disability [1], imposing a substantial burden on health care systems through its high incidence and the complex, prolonged care needs of survivors of stroke [2]. The effective management of stroke treatment and rehabilitation is limited by persistent challenges in postacute care, notably fragmented follow-up, insufficient community-based professional support, the heterogeneity of patient requirements, and frequently inadequate health literacy [3]. Despite significant progress in prevention strategies, acute treatments, and rehabilitation technologies, critical gaps persist in providing personalized, continuous, and accessible long-term support for individuals recovering from stroke [4]. These unmet needs highlight a critical opportunity for transformative technological innovation in the delivery and management of stroke care.

The analysis of clinical documentation presents an important strategic avenue for addressing stroke care challenges. Unstructured narratives within electronic health records, including clinical notes, discharge summaries, and other free-text entries, contain rich yet often underused patient information. Systematic analysis of these data can significantly support risk stratification, inform treatment planning, and improve care coordination [5]. This recognition has led to advancements in natural language processing (NLP) techniques designed to extract insights from complex clinical text. Fundamental to many clinical NLP applications are transformer-based models pretrained on extensive biomedical and general-domain corpora. Specifically, encoder-only architectures, which leverage bidirectional encoder representations from transformers and its derivatives, demonstrate proficiency in structured information extraction tasks such as named entity recognition [6] and temporal relation identification [7]. These models typically rely on domain-specific pretraining and task-specific fine-tuning. Nevertheless, they possess inherent limitations related to their generative capabilities and broader generalizability [8], with models often struggling with open-ended clinical reasoning tasks and understanding long contexts, indicating the need for architectures with enhanced generative potential.

Generative large language models (gLLMs), including decoder-only and encoder-decoder architectures (eg, the Llama [9], GPT-4 [10], and bidirectional and auto-regressive transformers [BART] [11] families), represent a significant advancement over previous NLP models. These gLLMs broaden clinical application possibilities by framing diverse tasks within a unified text generation paradigm [8,12]. Key enabling techniques include prompt-based learning, which enables task generalization without parameter updates [13], and inference-time controls (eg, decoding strategies) that modulate output characteristics, which are crucial when access to models is limited [14]. In addition, retrieval-augmented generation (RAG), often integrated with custom medical knowledge bases, enhances factual accuracy and performance for knowledge-intensive clinical applications [15,16]. Together, these advancements present important opportunities for stroke treatment and rehabilitation services [17], potentially improving efficiency through intelligent automation (eg, triage and administration); enhancing patient care through personalization and improved resource access; and accelerating research workflows, including evidence synthesis and writing. Furthermore, the introduction of multimodal functionality, as demonstrated by models such as GPT-4o [18] and the Gemini family [19], marks a pivotal shift in the development of gLLMs. By processing integrated textual, visual, and auditory inputs, these newly introduced models can augment clinical reasoning (eg, in medical image interpretation) and support more effective analysis of real-world, cross-modal patient data, better aligning digital tools with the complexities of stroke care delivery.

### Objectives

While digital health technologies provide advancements for stroke care [20], the unique capabilities and rapid evolution of gLLMs require a focused investigation within this specific clinical domain. Current reviews related to digital innovations in stroke care predominantly examine technologies that predate modern gLLMs, such as mobile health platforms [21,22], early conversational agents [23], and conventional machine learning or deep learning frameworks [24-26]. Moreover, although the current literature has reviewed the general clinical utility of gLLMs [17,27-29], there remains a critical gap in systematically reviewing evidence specifically on gLLM-driven interventions applied across the stroke care pathway. To address this critical research gap, this scoping review aimed to map the current landscape of gLLM applications throughout the common stages of the stroke care pathway. Specifically, it identified their uses, implementation characteristics, and reported outcomes and outlined future research directions. The central research question guiding this review was as follows: how, for what purposes, and with what reported outcomes have gLLMs been applied in stroke care? This review used the recommended guide of the Population, Concept, and Context (PCC) framework [30], which is guided by the following subquestions:

1. What study designs are used to evaluate gLLM-driven interventions in stroke care, and what are the key characteristics of the stroke populations involved? (Population or participants)
2. What target tasks, implementation details (ie, tasks, dialogue pattern, input data, and time stamps), evaluation approaches, and outcomes are reported for gLLM-driven interventions in stroke care? (Concept)
3. What cultural settings, specific stroke care stages (ie, prevention, diagnosis, treatment, prognosis, and rehabilitation), and technology adaptation strategies are described in the evaluation of gLLM-driven interventions? (Context)
4. What challenges are reported in implementing gLLMs in stroke care, and what specific directions for future research have been proposed? (Implementation challenges and research directions)

## Methods

### Study Guidelines and Registration

This review aimed to capture the available knowledge concerning the intersection of stroke care and gLLM technologies. Given the observed heterogeneity and breadth of research in this field, a scoping review methodology was used to summarize the current landscape and challenges associated with gLLM-driven intervention use across the stroke care pathway (ie, prediction, diagnosis, treatment, prognosis, and rehabilitation). The main objective was to address 3 key research questions predefined according to the PCC framework and identify knowledge gaps within this interdisciplinary area. This review was conducted and reported following the PRISMA-ScR (Preferred Reporting Items for Systematic Reviews and Meta-Analyses extension for Scoping Reviews) guidelines [31] (Multimedia Appendix 1) and adhered to the methodological framework of Arksey and O’Malley [32] for scoping reviews. The review protocol was preregistered on the Open Science Framework [33].

### Search Strategy

A broad search strategy was considered necessary to capture relevant citations in this relatively novel and rapidly evolving field. The terminology associated with gLLMs currently lacks consensus, requiring the use of diverse search terms. Key terms included in the search were “pretrained language model,” “large language model,” “natural language processing,” and “generative artificial intelligence.” Moreover, recognizing the important role of the generative pretrained transformer (GPT) model family in gLLM development, related terms were also incorporated into the search strategy. In addition, given the potential integration of gLLMs within conversational agents, relevant search terms for the latter were included to maximize retrieval breadth.

The search targeted peer-reviewed, full-text original research articles and was executed across 6 major scientific databases: Ovid Embase, PubMed, Scopus, CINAHL Plus with Full Text, Web of Science Core Collection, and IEEE Xplore. All database searches were completed in December 2024, with the last search performed on December 24, 2024. Search strategies were individually tailored to the syntax and indexing of each database. The complete search strategies for all databases are detailed in Multimedia Appendix 2. No restrictions regarding publication date, language, or study type were applied during the initial search phase. Potential selection bias arising from the absence of a standardized technical taxonomy or consensus definition for gLLMs was acknowledged as a limitation in this review. To mitigate this risk, snowballing techniques [34] were systematically used following the initial search. This involved both forward snowballing (ie, examining articles citing the included studies) and backward snowballing (ie, reviewing the reference lists of the included studies). However, this process did not identify any additional studies meeting this review’s inclusion criteria.

### Inclusion and Exclusion Criteria

To be eligible for this review, studies had to assess a gLLM-driven intervention relevant to advancing understanding or practice in stroke prediction, diagnosis, treatment, prognosis, or rehabilitation and report at least one metric or qualitative perspective related to the performance evaluation of the specified gLLM intervention. Studies were excluded if they met one or more of the following conditions: they (1) were animal trials or focused exclusively on animal models; (2) did not report any performance outcomes or evaluation pertinent to the gLLM intervention described; (3) were unrelated to the field of stroke care or its advancement; (4) focused exclusively on managing stroke risk factors (eg, diabetes mellitus, hypertension, or atrial fibrillation) without directly addressing stroke management, outcomes, or care processes; (5) had a full text that could not be accessed or obtained; or (6) did not represent original research (ie, were reflection articles, opinion pieces, editorials, letters, conference abstracts without full results, or study protocols).

### Study Selection and Data Extraction

Following the literature search, all retrieved records were imported into Zotero Reference Manager (version 7.0.15; Corporation for Digital Scholarship) by one author (XZ), where duplicates were identified and removed. Independent screening of titles, abstracts, keywords, and publication types was then conducted by 2 authors (XZ and WD) to identify potentially eligible studies based on the predefined inclusion criteria. The same 2 authors subsequently reviewed the full texts of these potentially eligible studies to confirm final inclusion and conduct data extraction (Multimedia Appendix 3). Any disagreements regarding study inclusion during either screening phase were resolved through discussion involving a third reviewer (ZL) until consensus was reached. Any unresolved issues encountered during feature extraction were documented as free-text notes; clarification was sought from the original study authors via email correspondence when necessary and feasible. All reviewers possessed relevant expertise in clinical medicine or medical informatics. Data extraction and synthesis activities were conducted in Microsoft Excel (Microsoft Office Long Term Service Channel 2021). Formal interrater agreement metrics were not calculated for the screening or extraction phases. This decision was made because the primary focus of this scoping review was the synthesis of descriptive characteristics, where minor formatting or phrasing differences between reviewers could lead to low numerical agreement despite substantive consensus on the content.

Guided by the PCC framework [35] and its predefined questions, the descriptive characteristics of the included studies were organized into structured tables. To confirm the methodological landscape and current evidence base at this emerging intersection of stroke care and gLLMs, this review commenced with a summary of study features, including publication year distribution and study design types. Then, consistent with the PCC framework, the analysis focused sequentially on (1) population (ie, characteristics relevant to intervention design and implementation, such as sample size, sex and age distributions, stroke phenotypes, and reported comorbidities); (2) concept (ie, key components describing the processes and outcomes of the gLLM-driven interventions, including the main use categories and specific tasks assigned to gLLMs; input data types used; dialogue patterns and time stamps recorded [where available]; and performance evaluation approaches based on reference standards, evaluative perspectives, and reported metrics); and (3) context, examining the broader cultural, care setting, and technical contexts surrounding the gLLM-driven interventions, including national and sociolinguistic backgrounds, the specific stage within the stroke care pathway addressed, models used, modes of gLLM access used, instruction design strategies, and other technical adaptations. Finally, key implementation challenges associated with applying gLLMs across the stroke care pathway were identified based on reported results and author discussions within the context of the included studies.

## Results

### Overview

**Figure 1:**
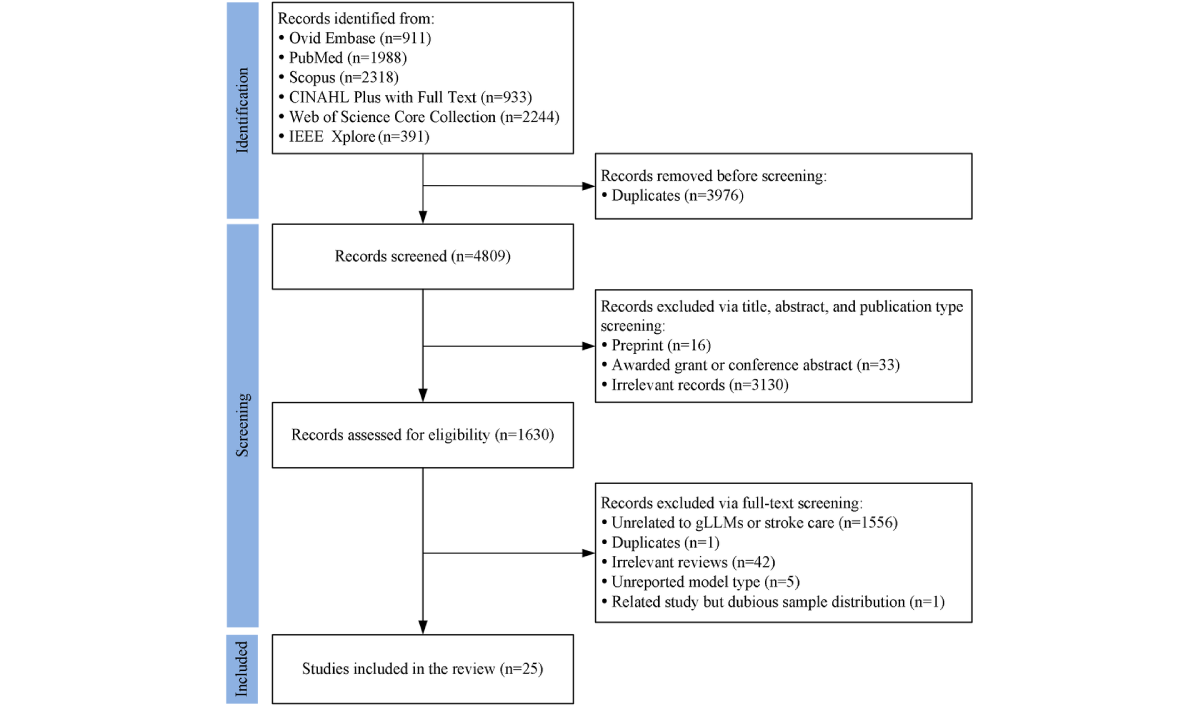
Flow diagram of the study selection process based on the PRISMA-ScR guidelines. gLLM: generative large language model.

The literature search identified 8785 records across all databases. Of these 8785 records, after the removal of 3976 (45.26%) duplicates, 4809 (54.74%) titles and abstracts were screened for eligibility. This initial screening led to the exclusion of 65.09% (3130/4809) of the records based on relevance and an additional 1.02% (49/4809) due to inappropriate publication types (eg, preprints, awarded grants, and conference abstracts). Consequently, 33.89% (1630/4809) of the articles underwent full-text assessment. During this stage, of the 1630 studies, 1605 (98.47%) were excluded for various reasons, including irrelevance to the application of gLLMs or stroke care context (n=1556, 96.95%), being review articles not meeting the inclusion criteria (n=42, 2.62%), insufficient evidence of gLLM use (n=5, 0.31%), being a duplicate publication identified across different formats (n=1, 0.06%), and unresolved concerns regarding stroke sample composition after author consultation (n=1, 0.06%). Ultimately, 25 studies met the inclusion criteria and were included in this scoping review. Figure 1 presents the detailed PRISMA-ScR flowchart illustrating this study selection process.

### General Characteristics

Table 1 summarizes the general characteristics of the 25 reviewed articles. A key characteristic was the recent publication time frame, with all included studies published in 2023 or 2024, reflecting the emerging nature of this research domain. With regard to the methodologies used, most studies (16/25, 64%) used retrospective designs analyzing existing data. A few studies (4/25, 16%) adopted prospective designs, typically involving the recruitment of healthy participants or the collection of original data from patients with stroke. There were also some observational studies (4/25, 16%), including one that used gLLMs for literature discovery during systematic review development [36], as well as a single comparative case study [37]. It should be noted that this review identified no randomized controlled trials assessing the clinical efficacy or impact of gLLM-driven interventions in populations of patients with stroke.

**Table 1:** Overview of study designs and stroke populations.

Study	Year	Study design	Sample size	Sex (male; %)	Age (y)	Stroke phenotype	Comorbidities	Function scoring tool
Pedro et al [38]	2025	Retrospective; pilot	163	39.3	Mean 74 (SD 18)	IS^a^	AF^b^, HF^c^, HTN^d^, DM^e^, DLP^f^, CAD^g^, and AC^h^	NIHSS^i^: 14.0 (9.0); ASPECTS^j^: 9.0 (2.0); mRS^k,l^
Chen et al [39]	2024	Retrospective	124 (22 simulated)	NR^m^	Median 66 (IQR NR)^n^	IS and HS^o^	HTN, HF, and ESRD^p^	NIHSS: median 12 (IQR NR)^n^; mRS: median 1 (IQR NR)^n^
Strotzer et al [40]	2024	Retrospective	Uncertain^q^	NR	NR	IS and HS	NR	NR
Kuzan et al [41]	2025	Retrospective	Uncertain^r^	NR	NR	IS	NR	NR
Fei et al [42]	2024	Prospective; cross-sectional	30^s^	60	68.03 (3.74)	Unclarified type	NR	NR
Lee et al [43]	2024	Retrospective	46	63.1	56.7 (13.9)	IS and HS	HTN, DM, DLP, AF, CAD, and other^t^	NIHSS, mRS, MRC^u^ Scale for Muscle Strength, GCS^v^, K-MMSE^w^, FAB^x^, and other^t^
Haim et al [44]	2024	Retrospective	30	NR	NR	Unclarified type	NR	NR
Chen et al [45]	2023	Experimental	20 simulated	50	65.3 (11.0)	HS	NR	GCS: 12.5 (5); ICH^y^ score: 2 (2); H&H^z^: 2.5 (2)
Blacker et al [46]	2024	Observational	2 simulated	50	70.5 (4.5)	IS	AF and AC	NR
Zhang et al [37]	2023	Observational; comparative case	1 textbook case	100	62 (—^aa^)	IS	DM and HTN	SIAS^ab,ac^
Sivarajkumar et al [47]	2024	Retrospective	13,605^ad^	49	75 (16)	Unclarified type	NR	NR
Guo et al [48]	2023	Retrospective	Uncertain^ae^	—	—	IS and HS	NR	NR
Lehnen et al [49]	2024	Retrospective	130 (derivation: 100; external validation: 30)	50	74.2 (13.2)	IS	NR	NIHSS: median 8 (IQR 0-24); ASPECTS: median 9 (IQR 3-10)^af^
Fiedler et al [50]	2024	Retrospective; pilot	50	62	Median 4.5 (IQR 0.75-11)	CAIS^ag^, PAIS^ah^, and CVST^ai^	NR	PSOM^aj^: median 0.75 (IQR 0-1.5)
Wang et al [51]	2024	Retrospective	382	54.45	72.23 (13.35)	IS	NR	NR
Goh et al [52]	2024	Retrospective	16	37.5	76.1 (11.4)	IS	AF, DM, and HTN	NR
Baro et al [53]	2025	Retrospective	Uncertain^ak^	NR	NR	Unclarified type	NR	NR
Meddeb et al [54]	2025	Retrospective	Uncertain^al^	NR	NR	IS	NR	NR
Kim et al [55]	2025	Retrospective	36,922	58.8	68.17 (12.86)	IS	HTN, AF, DM, DLP, CAD, AC, PAD^am^, and HF	NIHSS: 2 (5); mRS: 2 (3)
Argymbay et al [56]	2024	Retrospective	4798	65.1	47.1 (23.7)	IS	HTN, DM, DLP, and obesity	NIHSS: 18.1 (11.3); mRS: 3.7 (1.9)
Neo et al [57]	2024	Prospective; mixed methods	50	NR	NR	Unclarified type	NR	NR
Wu et al [58]	2023	Observational	—^an^	—	—	—	—	—
Chen et al [59]	2025	Prospective; experimental	1^ao^	NR	NR	Unclarified type	NR	NR
Rifai et al [60]	2024	Prospective; experimental	Uncertain^ap^	NR	NR	Unclarified type	NR	NR
Anghelescu et al [36]	2023	Observational	—^aq^	—	—	—	—	—

^a^IS: ischemic stroke.

^b^AF: atrial fibrillation.

^c^HF: heart failure.

^d^HTN: hypertension.

^e^DM: diabetes mellitus.

^f^DLP: dyslipidemia.

^g^CAD: coronary artery disease.

^h^AC: active cancer.

^i^NIHSS: National Institutes of Health Stroke Scale.

^j^ASPECTS: Alberta Stroke Program Early Computed Tomography Score.

^k^mRS: modified Rankin Scale.

^l^A total of 121 patients had an mRS score of 0 or 1, and 42 had a score of 2 or 3.

^m^NR: not reported.

^n^Description of real patients (n=102).

^o^HS: hemorrhagic stroke.

^p^ESRD: end-stage renal disease.

^q^A total of 100 magnetic resonance and computed tomography images were included, comprising 50 with lesions (25 ischemic stroke, 25 brain hemorrhage) and 50 normal controls (25 matched to each lesion group).

^r^A total of 266 radiological images from patients with acute stroke were included.

^s^A total of 90 participants were included, comprising 30 patients with stroke and 60 healthy controls.

^t^Published case report heterogeneity led to reporting barriers.

^u^MRC: Medical Research Council.

^v^GCS: Glasgow Coma Scale.

^w^K-MMSE: Korean version of the Mini-Mental State Examination.

^x^FAB: Frontal Assessment Battery.

^y^ICH: intracranial hemorrhage.

^z^H&H: Hunt and Hess scale.

^aa^Not applicable.

^ab^SIAS: Stroke Impairment Assessment Set.

^ac^A multicriteria assessment set included quantitative scores and qualitative descriptions.

^ad^In total, 50 annotated electronic health record sections were extracted from the records of 13,605 patients with stroke.

^ae^Parts from triplets, subrelations, and unlabeled text from 3 Chinese stroke-related medical datasets were included.

^af^Description of derivation (n=100).

^ag^CAIS: childhood arterial ischemic stroke.

^ah^PAIS: perinatal arterial ischemic stroke.

^ai^CVST: cerebral venous sinus thrombosis.

^aj^PSOM: Pediatric Stroke Outcome Measure.

^ak^At least 4038 stroke-related hospitalizations of insured beneficiaries were included in the study.

^al^A total of 1050 mechanical thrombectomy reports from patients with acute ischemic stroke were included.

^am^PAD: peripheral arterial disease.

^an^Two questions from the American Stroke Association website were included.

^ao^Three healthy participants were also involved in the test of the generative large language model–based hand exoskeleton controls.

^ap^Did not report whether the 12 participants were patients with stroke.

^aq^Six questions on evidence synthesis during systematic reviews were included.

### Distribution of Included Stroke Populations

The first question of this review related to the *population* component of the PCC framework and asked for key characteristics of the stroke populations involved in the gLLM-driven interventions. Specifically, the review examined the characteristics of the stroke populations involved in the included studies. The analysis included sample size, sex distribution, age range, stroke phenotypes, key comorbidities, and reported functional scores as these elements can influence intervention design and applicability. Notably, 8% (2/25) of the studies did not use patient data (real or simulated); instead, they evaluated the gLLMs using predefined question sets related to stroke care [36,57]. Among the remaining 92% (23/25) of the studies, the level of detail provided for population characteristics varied. A summary of these characteristics, including clarifications obtained via author correspondence, is presented in Table 1.

Reporting of specific population characteristics varied across the 25 studies (see Table 1 for further details). Sample sizes of involved patients were specified in most articles (17/25, 68%), demonstrating considerable range from a single case to 36,922 patients. Data on gender were available in 52% (13/25) of the studies, which indicated that male individuals comprised 56.9% of the aggregate reported sample. A total of 56% (14/25) of the studies provided age metrics (mean or median), which spanned 4.5 years (in a pediatric study) to 76.1 years. Stroke phenotype details were available in 64% (16/25) of the studies, and ischemic stroke (15/25, 60%) was found to be more commonly studied than hemorrhagic stroke (5/25, 20%). Notably, 4% (1/25) of the studies focused exclusively on pediatric patients with stroke. In total, 32% (8/25) of the studies provided information on patient comorbidities, often identified through the main text, appendices, or associated datasets. Commonly reported conditions included hypertension, diabetes mellitus, atrial fibrillation, dyslipidemia, coronary artery disease, heart failure, and active cancer. Furthermore, 36% (9/25) of the studies documented baseline severity or functional outcomes using clinical assessment tools. The most frequently used scales were the National Institutes of Health Stroke Scale and the modified Rankin Scale [38,39,43,55,56]. Other reported instruments included the Glasgow Coma Scale [43,45], Pediatric Stroke Outcome Measure [50], Stroke Impairment Assessment Set [37], intracranial hemorrhage score [45], Hunt and Hess scale [45], Medical Research Council Scale for Muscle Strength [43], Korean version of the Mini-Mental State Examination [43], and Frontal Assessment Battery [43].

### Conceptual Considerations for Implementing and Evaluating gLLM-Driven Interventions in Stroke Care

In response to the second subquestion related to the *concept* component of the PCC framework, this review analyzed the target tasks, implementation details (including models, prompts, and data inputs), evaluation strategies, and reported outcomes for gLLM applications in stroke care. In total, 4 key categories of gLLM use were identified, as summarized in Table 2. The main categories focused on supporting health care professionals either through clinical decision-making assistance (10/25, 40%) or administrative workflow automation (9/25, 36%). Other identified applications included direct patient support through interactive online platforms (5/25, 20%) and enabling the discovery of evidence during systematic reviews (1/25, 4%). With regard to the implementation of gLLMs, evaluations mostly involved single-turn dialogues conducted under controlled settings (15/25, 60%), whereas the reporting of intervention time stamps was limited (5/25, 20%). Despite considerable heterogeneity across studies in terms of task objectives, input data sources, evaluation benchmarks, and assessment metrics, common themes and approaches were found within each application category.

**Table 2:** Summary of the implementation and evaluation of generative large language model–driven interventions in stroke care.

Study		Task objectives	Input data or sources	Dialogue patterns	Reported time stamp	Gold-standard providers or benchmarks	Evaluation perspectives	Evaluation metrics
**Clinical decision-making support (n=10)**								
	Pedro et al [38]	Predict the mRS^a^ score at 3 mo after mechanical thrombectomy	Patient H&P^b^, neuroimaging, and mechanical thrombectomy procedure notes	Single turn	Yes	Stroke unit clinicians	AGS^c^ for true exact and dichotomized mRS scores; bias; comparison with MT-DRAGON	Cohen κ; mean difference and 95% limits of agreement; ND^d^
	Chen et al [39]	Make clinical decisions for mechanical thrombectomy	Patient H&P and neuroimaging notes	Single turn	No	Neurology specialists	AGS for mechanical thrombectomy decision; different error analysis	Counts and rate
	Strotzer et al [40]	Interpret MRI^e^ and CT^f^ images and generate free-text reports in stroke cases	MRI and CT images	Single turn	Yes	Radiologists and nonradiologist in training	AGS for free-report items; interrun consistency; AGS for binary pathological findings; impact on nonradiologist	Agreement rate; interrun consistency rate and the Randolph free-marginal κ; accuracy, sensitivity, and specificity; rate (distribution across categories)
	Kuzan et al [41]	Interpret DWI^g^ and ADC^h^ maps in acute stroke cases	DWI and ADC maps	Multiturn	No	Radiologists	AGS for stroke and normal or all-image interpretation	Rate; TP^i^, TN^j^, FP^k^, FN^l^, sensitivity, specificity, PPV^m^, NPV^n^, and accuracy
	Fei et al [42]	Evaluate cognitive performance in stroke cases	Patient responses to selected RBMT-II^o^, MMSE^p^, and MoCA^q^ items	Multiturn	No	Rehabilitation physicians	Intermodel and model-physician agreement	Intraclass correlation coefficient and *P* value
	Lee et al [43]	Locate lesions based on patient H&P	Patient H&P notes	Single turn	Yes	Location description from original published case report	AGS for trial- and case-based lesion localization; different error analysis	Specificity, sensitivity, precision, and *F*_1_-score; ND
	Haim et al [44]	Calculate the NIHSS^r^ score and predict the use of tissue plasminogen activator	EMR^s^ periods	Single turn	No	Emergency department physicians	Intermodel and model-physician agreement; predictive validity	Cohen κ and *P* value; AUC-ROC^t^
	Chen et al [45]	Calculate GCS^u^, H&H^v^, and ICH^w^ scores	Patient neuroexamination notes without scores	Single turn	No	Scores in original neuroexamination notes	AGS for scoring; repeatability; effect of varied case complexity and prompting design	Average error rate and average error magnitude
	Blacker et al [46]	Use of SNACC^x^ HQRs^y^ to answer questions on perioperative stroke and endovascular treatment anesthesia	Patient H&P notes	Multiturn	Yes	Anesthesiologists	HQR identification; correct reference citation; potentially harmful information	ND
	Zhang et al [37]	Generate rehabilitation prescriptions and ICF^z^ codes in a stroke case	Patient H&P notes	Multiturn	No	Physical medicine and rehabilitation physicians	Content exhaustiveness and clinical applicability; inference logic	ND
**Administrative assistance (n=9)**								
	Sivarajkumar et al [47]	Extract and categorize physical rehabilitation exercise information from stroke cases	EHR^aa^ sections with physical therapy information	Single turn	No	Physical therapy experts	AGS for extracted items	Accuracy, precision, recall, and *F*_1_-score
	Guo et al [48]	Extract triples by fine-tuning and integrating a relation classification module	Stroke-related medical text from SEMRC^ab^, CVDEMRC^ac^, and CMeIE^ad^	—^ae^	No	Relevant items from datasets and performance of the Cas-CLN^af^ benchmark models	AGS for total and overlapping triple extraction; performance improvements over baseline models	*F*_1_-score; rate
	Lehnen et al [49]	Extract key information for mechanical thrombectomy	Mechanical thrombectomy records	Single turn	No	Interventional neuroradiologists	AGS for extracted items; different error analysis; intermodel extraction performance comparison	Correct rate and Cohen κ; count and rate; correct rate and *P* value
	Fiedler et al [50]	Extract IPSS^ag^ format information and infer disease severity	Outpatient notes	Multiturn	No	Clinical investigators	AGS for extracted items	Rate
	Wang et al [51]	Extract and infer key information for mechanical thrombectomy surgery	Mechanical thrombectomy records	Single turn and multiturn for correct format response	No	Interventional and junior neuroradiologists	AGS for extracted and inferred items; agreement with junior neuroradiologists; processing efficiency	Accuracy, sensitivity, specificity, AUC^ah^, and mean squared error; *P* value; average case processing time
	Goh et al [52]	Extract stroke audit data	Discharge summaries	Single turn	No	Relevant items from original discharge summaries	AGS for extracted items; model-clinician comparison in AGS; inference error analysis	Counts and rate; ND
	Baro et al [53]	Predict stroke hospitalization by fine-tuning and integrating classification layers	Chronological health insurance data with aggregated medical events	—	No	Relevant items from original health insurance data	AGS across time windows using the general fine-tuned models; AGS comparison between general and stroke-specific fine-tuned models	*F*_1_-score, sensitivity, specificity, and AUC
	Meddeb et al [54]	Extract key information for mechanical thrombectomy items	Mechanical thrombectomy records	Single turn	No	Radiologists and clinical medical students	AGS for extracted items; efficiency improvement with EITL^ai^	Precision, recall, and *F*_1_-score; average case time savings
	Kim et al [55]	Perform data wrangling on a large dataset of patients with stroke	Metadata from the CRCS-K^aj^ dataset and neurologist queries	Multiturn	No	Neurologists	Reliability and efficiency of EITL workflow and clinical knowledge alignment	ND
**Direct patient interaction (n=5)**								
	Argymbay et al [56]	Provide personalized stroke risk insights and answer medical queries based on patient data	Stroke risk values, medical literature, and patient queries	Multiturn	No	Clinicians	Stroke risk factor review, personalized health recommendation provision, and anxiety alleviation	ND
	Neo et al [57]	Answer rehabilitation questions for patients with stroke and their caregivers	280 unique questions	Single turn	Yes	Clinicians	Content correctness, safety, relevance, and readability; interrater agreement; free comments for responses	3-point Likert scale; Fleiss κ and Cohen κ; ND
	Wu et al [58]	Provide nonmedical professionals with stroke-related health information	2 questions about stroke prevention from the ASA^ak^ website	Single turn	No	Answers available on the ASA website	Readability compared with the Google Assistant; content relevance	Word counts, GFS^al^, SMOG^am^ index, DCS^an^, FKRT^ao^, and *P* value; keyword matching counts
	Chen et al [59]	Interpret commands and generate Python code for hand exoskeleton control	Recognized user voice commands	Single turn	No	Rehabilitation physicians	Executability and efficiency of tasks among models; response process in free scenarios	Success rate across trials and time; ND
	Rifai et al [60]	Interpret commands and generate target coordinates for upper-limb robot control	Recognized user voice commands	Single turn	No	Predefined targets	Executability of path to targets compared with joystick control; intuitive handling; success and stable control	ND; user experience questionnaire; success rate across trials and ND
**Automated literature review (n=1)**								
	Anghelescu et al [36]	Assist in obtaining evidence on Actovegin’s efficacy for ischemic stroke	6 queries on medicine, review conduction, literature exploration, and evidence synthesis	Multiturn	No	Review contributors	General and in-depth answer correctness; citation applicability; PRISMA^ap^-based evidence synthesis results	ND

^a^mRS: modified Rankin Scale.

^b^H&P: history and neurological physical examination.

^c^AGS: agreement with the gold standard.

^d^ND: narrative description.

^e^MRI: magnetic resonance imaging.

^f^CT: computed tomography.

^g^DWI: diffusion-weighted imaging.

^h^ADC: apparent diffusion coefficient.

^i^TP: true positive.

^j^TN: true negative.

^k^FP: false positive.

^l^FN: false negative.

^m^PPV: positive predictive value.

^n^NPV: negative predictive value.

^o^RBMT-II: Rivermead Behavioral Memory Test–II.

^p^MMSE: Mini-Mental State Examination.

^q^MoCA: Montreal Cognitive Assessment.

^r^NIHSS: National Institutes of Health Stroke Scale.

^s^EMR: electronic medical record.

^t^AUC-ROC: area under the receiver operating characteristic curve.

^u^GCS: Glasgow Coma Scale.

^v^H&H: Hunt and Hess scale.

^w^ICH: intracranial hemorrhage.

^x^SNACC: Society for Neuroscience in Anesthesiology and Critical Care.

^y^HQR: high-quality recommendation.

^z^ICF: International Classification of Functioning, Disability, and Health.

^aa^EHR: electronic health record.

^ab^SEMRC, stroke EMR entity and entity-related corpus.

^ac^CVDEMRC: cardiovascular EMR entity and entity relationship–labeling corpus.

^ad^CMeIE: Chinese Medical Information Extraction dataset.

^ae^Not applicable.

^af^Cas-CLN: cascade binary pointer tagging network with conditional layer normalization.

^ag^IPSS: International Pediatric Stroke Study.

^ah^AUC: area under the curve.

^ai^EITL: expert in the loop.

^aj^CRCS-K: Clinical Research Collaboration for Stroke in Korea.

^ak^ASA: American Stroke Association.

^al^GFS: Gunning fog score.

^am^SMOG: Simple Measure of Gobbledygook.

^an^DCS: Dale-Chall score.

^ao^FKRT: Flesch-Kincaid readability test.

^ap^PRISMA: Preferred Reporting Items for Systematic Reviews and Meta-Analyses.

gLLM-driven systems categorized as clinical decision-making support were mainly used to analyze clinical documentation to inform medical diagnosis, treatment planning, prognosis estimation, or rehabilitation strategies in stroke care. While textual inputs such as the medical history of patients, neurological examination results, and neuroimaging reports were common, only 8% (2/25) of the studies analyzed computed tomography or magnetic resonance imaging scans directly as primary input [40,41]. Such gLLM-driven systems were applied across the stroke care pathway, assisting with neurological function scoring during triage (eg, the National Institutes of Health Stroke Scale [44], Glasgow Coma Scale, Hunt & Hess scale, and intracranial hemorrhage score [45]) and supporting diagnosis through direct image interpretation [40,41] or lesion mapping from textual descriptions [43]. In addition, they were used to inform acute intervention decisions, including eligibility for thrombectomy [39] or thrombolysis [44] and anesthesia planning [46]. Moreover, they facilitated rehabilitation through outcome prediction (eg, 3-month modified Rankin Scale [38]), cognitive assessment [42], or generation of personalized rehabilitation plans [37]. Performance evaluation mainly involved clinician assessment or comparison against predefined benchmarks derived from the original clinical records. Additional validation methods often included cross-comparison against the outputs of clinicians or functionally similar tools using identical inputs [38,39,42,44], as well as repeatability checks across multiple models [40,43,45]. Some studies (5/25, 20%) investigated human-computer interaction factors, examining aspects such as the impact on junior clinicians [40] or examining the reasoning processes behind model-generated conclusions [37,39,43,46]. Across these varied approaches, quantitative metrics (eg, accuracy, rate, *F*_1_-score, *k* value, and *P* value), particularly those assessing factual accuracy and output consistency, were the primary focus of most evaluations.

gLLM-driven systems categorized as administrative support predominantly focused on alleviating clinician documentation workload and improving the management and use of clinical information. The primary functions involved extracting structured information from clinical text and generating summaries or other abstract representations to facilitate downstream use by other health care workers. These tasks used a variety of clinical data sources, including electronic health records [47], electronic medical records [48], specialized procedural records (eg, thrombectomy reports) [49,51,54], discharge summaries [52], outpatient notes [50], health insurance claim data [53], and stroke registries [55]. Evaluation methods for these administrative tasks were similar to those used for decision support tools. Most often, the alignment of gLLM outputs with gold-standard annotations was measured [47-55], or performance was compared against that of human experts or other specialized systems that were used to analyze identical data [48,49,51-53]. Quantitative metrics were used most frequently during performance assessments [47-54]. Beyond accuracy and alignment, a few studies (3/25, 12%) explicitly evaluated efficiency. For example, 4% (1/25) of the studies reported the average time required for automated data extraction from thrombectomy operative notes [51], whereas another 8% (2/25) demonstrated significant time reductions using expert-in-the-loop (EITL) workflows involving gLLMs for extracting procedural details [54] and processing large-scale registry data [55].

gLLM-driven systems involving direct patient interaction were developed primarily to support personalized out-of-hospital stroke care, reduce patient uncertainty regarding medical information, and promote adherence to preventive and rehabilitative behaviors. The main tasks performed by gLLMs in this regard included (1) answering general stroke-related queries using embedded knowledge [57,58], (2) generating individualized preventive guidance by interpreting patient profiles with relevant literature [56], and (3) translating natural language commands to control upper-limb exoskeleton robots during rehabilitation [59,60]. Consequently, study designs focused on addressing patient needs, either through simulating responses to public-facing queries [57,58] or by developing systems intended specifically for lay users [56,59,60]. Assessment strategies for these systems considered both technical output performance (eg, factual alignment [56-60] and comparative analyses against alternative methods [59,60]) and key patient-centered outcomes. The latter included metrics such as readability [57,58], safety [57], personalized support [57,58], potential for anxiety reduction [56], and overall user experience [60]. As a result, the open-ended and dialogue-driven nature of these systems required diverse evaluation methodologies. These ranged from clinician-led narrative assessments or reviews [56,57,59,60] and independent scoring protocols [57] to user feedback questionnaires [60] and standard quantitative metrics computed by the research teams [58-60].

Only 4% (1/25) of the included studies [36] investigated the application of gLLM systems to support literature review tasks. This study involved asking 6 questions to the gLLM, ranging from general medical knowledge and systematic review methodology inquiries to specific queries about evidence synthesis concerning Actovegin’s efficacy for ischemic stroke. A qualitative evaluation of the gLLM-generated answers assessed their correctness and applicability for the review context. The study concluded that all responses generated by the gLLM were unreliable, resulting in their exclusion from the final systematic review conducted by the research team. Table 2 provides a summary of the target tasks, implementation characteristics, and evaluation approaches reported across the included studies.

### Contextual Focus on gLLM-Driven Intervention Design in Stroke Care

In response to the third subquestion and the *context* component of the PCC framework, this review examined the settings surrounding the design and implementation of the evaluated gLLM interventions, with further information presented in Table 3. This review considered 3 primary contextual dimensions: cultural, care, and technical settings. Cultural context referred to the study location (country) and relevant national and sociolinguistic backgrounds of the participants (eg, health care professionals, patients, and caregivers). The care dimension referred to the specific phase of the stroke care pathway (ie, prevention, diagnosis, treatment, prognosis, or rehabilitation) targeted by the intervention and associated data sources. The technical dimension involved the diverse adaptation choices evident in intervention development, including approaches used in instruction design (prompt engineering), inference-time parameter configurations, and underlying model-level adaptations.

**Table 3:** Summary of generalized large language model–driven intervention design in stroke care.

Study	Country	Stage in the stroke care continuum	Foundation model or model series	Access	Instruction design	Other adaptation strategies
Pedro et al [38]	Portugal	Prognosis	GPT-3.5	Web-based chat interface (ChatGPT)	Zero shot	None
Chen et al [39]	United States	Treatment	GPT-4	Web-based chat interface (ChatGPT)	Zero shot, role based, context enhanced, and format constrained	None
Strotzer et al [40]	Germany	Diagnosis	GPT-4-1106-vision-preview	Official API^a^ (via OpenAI platform)	Zero shot, role based, context enhanced, and format constrained	None
Kuzan et al [41]	Turkey	Diagnosis	GPT-4 Vision	Web-based chat interface (ChatGPT)	Zero shot and context enhanced	None
Fei et al [42]	China	Rehabilitation	GPT-3.5 and GPT-4	Web-based chat interface (ChatGPT)	Zero shot, role based, and context enhanced	None
Lee et al [43]	—^b^	Diagnosis	GPT-4	Unclarified	Zero shot, chain of thought, context enhanced, and format constrained	None
Haim et al [44]	Israel	Diagnosis and treatment	GPT-3.5 and GPT-4	Web-based chat interface (ChatGPT)	Zero shot and context enhanced	None
Chen et al [45]	United States	Diagnosis	GPT-4	Web-based chat interface (Bing chat)	Zero shot, role based, and context enhanced	None
Blacker et al [46]	United States	Treatment	GPT-4	Web-based chat interface (ChatGPT)	Zero shot and context enhanced	None
Zhang et al [37]	Japan	Rehabilitation	GPT-4	Web-based chat interface (ChatGPT)	Zero shot	None
Sivarajkumar et al [47]	United States	Rehabilitation	GPT-3.5-turbo	Official API (via Microsoft Azure)	Zero shot, few shot, role based, and format constrained	None
Guo et al [48]	China	Diagnosis and treatment	BART^c^-base-Chinese and BART-large-Chinese	Unclarified	—	Fine-tuning, constrained decoding, encoding representation reuse, beam search, feature fusion, and shared encoder weights
Lehnen et al [49]	Germany	Treatment	GPT-3.5 and GPT-4	Web-based chat interface (ChatGPT)	Zero shot, format constrained, and context enhanced	None
Fiedler et al [50]	United States	Diagnosis, treatment, prognosis, and rehabilitation	GPT-3.5-turbo-16k	Official API (via Microsoft Azure)	Zero shot, role based, format constrained, and context enhanced	Temperature set to 0
Wang et al [51]	China	Treatment	GPT-3.5-turbo, GPT-4, Gemini Pro, GLM-4, Spark 3, and Qwen-Max	Official APIs (via unclarified platforms)	Zero shot, format constrained, and context enhanced	None
Goh et al [52]	Australia	Diagnosis and treatment	Llama 3-70B	Local inference	Zero shot, role based, and format constrained	Temperature set to 0
Baro et al [53]	Brazil	Prevention	openCabrita 3B	Unclarified	—	Low-rank adaptation tuning
Meddeb et al [54]	Germany	Treatment	Qwen-72B, Mixtral 8x7B, and BioMistral-7B	Local inference	Zero shot, format constrained, and context enhanced	None
Kim et al [55]	South Korea	Diagnosis, treatment, and prognosis	GPT-4-32k	Official API (via unclarified platform)	Few shot, format constrained, and context enhanced	Low-temperature setting
Argymbay et al [56]	Canada	Prevention	BioMistral-7B	Private API (via Hugging Face on Amazon SageMaker)	Few shot and context enhanced	Temperature set to 0.3
Neo et al [57]	Singapore	Rehabilitation	GPT-3.5-turbo and PaLM 2	Web-based chat interfaces (ChatGPT and Google Bard)	Zero shot and context enhanced	None
Wu et al [58]	United States	Prevention	GPT-3.5	Web-based chat interface (ChatGPT)	Zero shot	None
Chen et al [59]	China	Rehabilitation	GPT-4, GPT-3.5-turbo, and GLM-130B	Official APIs (via unclarified platforms)	Few shot, role based, format constrained, and context enhanced	None
Rifai et al [60]	Indonesia	Rehabilitation	GPT-4o	Official API (via unclarified platforms)	Zero shot, format constrained, and context enhanced	Temperature set to 0.5; token generation minimized
Anghelescu et al [36]	—	Treatment	Unclarified GPT^d^	Web-based chat interface (ChatGPT)	Zero shot	None

^a^API: application programming interface.

^b^Not applicable.

^c^BART: bidirectional and auto-regressive transformers.

^d^GPT: generative pretrained transformer.

Analysis of the cultural dimension identified the geographic settings for most of the included studies (23/25, 92%). The studies originated from diverse global locations, with the United States (6/25, 24%), China (4/25, 16%), and Germany (3/25, 12%) being the most represented countries. Other studies represented individual contributions from Canada, Australia, Singapore, Japan, South Korea, Turkey, Portugal, Brazil, Indonesia, and Israel. With regard to the care dimension, most gLLM interventions (11/25, 44%) focused on the treatment phase, where systems were typically used to support clinical decisions, integrate therapeutic guidelines, or extract specific treatment data (eg, surgical procedures and medication regimens) from documentation. The diagnostic phase was the second most common focus (9/25, 36%), with applications including lesion localization support, assistance with diagnostic reasoning, and extraction of pertinent diagnostic details from clinical records. Considerably fewer studies focused on stroke prevention (3/25, 12%) or prognosis (3/25, 12%). Prevention-focused interventions mainly aimed to reduce subsequent stroke-related hospitalizations or expand public access to preventive resources. Prognostic applications focused on assisting clinicians primarily by calculating prognostic scores or interpreting relevant information documented within clinical notes.

Regarding the technical dimension, adaptation strategies for the gLLM-driven systems varied across the included studies. These choices often reflected trade-offs between computational cost and task demands, aiming to align model behavior with task-specific constraints while maintaining stable output control. For relatively straightforward tasks, a *plug-and-play* strategy using standard interfaces was frequently adopted. This involved accessing closed-source models using web-based chat interfaces (12/25, 48%) or application programming interface (API) end points (8/25, 32%) without further model customization. As task complexity increased or baseline performance proved inadequate, studies often adopted multiprompt strategies to better guide model behavior. Established methods included zero-shot (20/25, 80%), few-shot (4/25, 16%), and chain-of-thought (1/25, 4%) prompting. Beyond these approaches, specific prompting techniques were used to improve control—role-based prompting assigned domain-specific personas (eg, *You are a neurologist*); format-constrained prompting enforced structured outputs (eg, JSON, CSV, standardized terminologies, and executable code); and context-enhanced prompting incorporated background knowledge, task decomposition steps, or self-reflection instructions to improve response quality.

These prompting strategies were sometimes used alongside inference-time configurations, among which temperature adjustment was the most frequently reported technique (5/25, 20%) for modulating output diversity versus coherence. In a small subset of studies requiring deeper customization (2/25, 8%), locally deployed open-source models underwent model-level adaptations. These included techniques such as parameter-efficient fine-tuning and architectural modifications to customize the model more closely to the specific clinical application. A variety of gLLM families were used across the included studies. The GPT series (OpenAI) was mainly used in 80% (20/25) of the studies. Other models used in multiple studies included the Mixtral (and its variant, BioMistral) series (Mistral AI; 3/25, 12%), the PaLM 2 (and its successor, Gemini) series (Google DeepMind; 2/25, 8%), the Qwen series (Alibaba Cloud; 2/25, 8%), and the GLM series (Zhipu AI; 2/25, 8%). Models identified in single studies included Llama 3-70B (Meta), BART base and BART-large-Chinese (Fudan NLP Lab), Spark 3 (iFLYTEK), and openCabrita 3B (22h).

### Challenges Identified During the Implementation of gLLM-Driven Interventions in Stroke Care

Through a comprehensive review of the findings of the included studies, five key challenges were identified in applying gLLMs across the stroke care pathway: (1) ensuring factual alignment, (2) maintaining system robustness, (3) enhancing model interpretability, (4) optimizing operational efficiency, and (5) facilitating adoption into clinical practice.

Factual alignment was the most frequently discussed concern [36-55,57-60], reflecting persistent difficulties in ensuring consistency among system outputs, established clinical knowledge, and input data. Documented issues included inaccurate or incomplete responses, hallucinated content, and output failures. Several studies (11/25, 44%) noted nondeterministic behavior across repeated runs [41,43,45,51], failure to retrieve pretrained knowledge [36,40,46,57], limited inclusion of up-to-date evidence [37,38], and inconsistencies between the model’s reasoning steps and its final outputs [39,43]. Robustness issues were mainly associated with variability in output quality due to changes in input data or instructions. Data-related concerns included difficulty in handling rare or complex cases [38-41,43,45,50,54,55,59,60]; managing human-induced input noise such as incompleteness, ambiguity, or internal contradiction [38-40,44,45,49,51,52,54]; and adapting to distributional discrepancies between training and deployment data [40,41,43,48,57]. Instruction-level fragility was also observed as small prompt modifications led to substantial variations in output [37,40,42,43,46,47,49,50,54,58], demonstrating the sensitivity of gLLM-driven systems to prompt design.

Adoption, interpretability, and efficiency were also deemed potential concerns in applying gLLMs across the stroke care pathway. Adoption-related challenges involved the need for EITL oversight when applying gLLMs [36,37,39-41,44,45,49,50,52,58]; ongoing efforts to integrate gLLMs into clinical workflows [40,42,43,45,50,52,55,57,60]; and unresolved issues related to legal compliance, data privacy, and patient safety [43,50,57]. Interpretability challenges were associated with the opaque and uncontrollable nature of gLLM reasoning [41,43,46,55,57], the limited readability of gLLM responses [57,58], and variations in how individuals understood the same content [46,57]. Efficiency-related concerns included token processing constraints [38,39] and trade-offs between model performance and computational cost [53,59].

## Discussion

### Principal Findings

**Figure 2:**
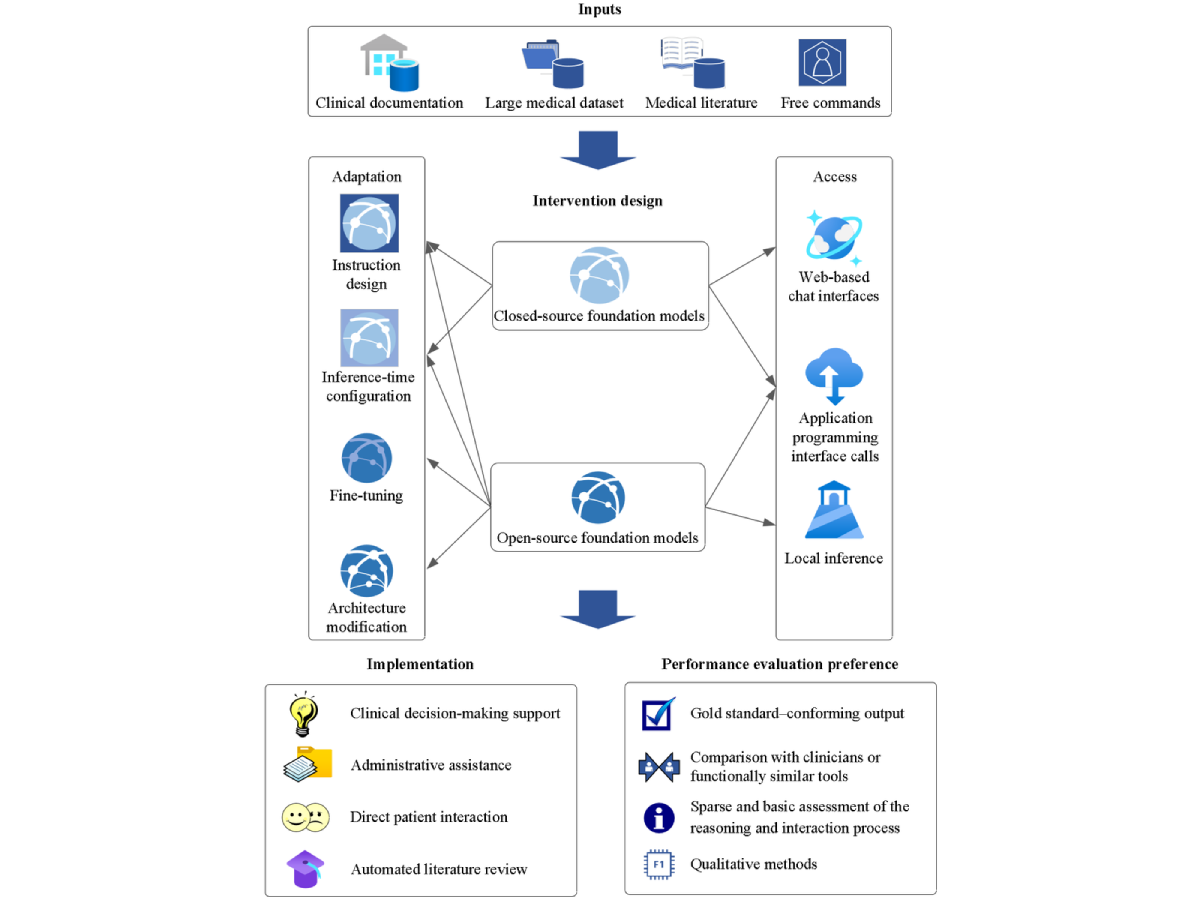
Current landscape of interventions driven by generative large language models in stroke care.

This study presented a timely scoping review mapping the intersection of stroke care and gLLMs, providing practical insights into current applications within this rapidly evolving domain. The substantial heterogeneity identified across the included studies, spanning objectives, methodologies, contexts, and outcomes, precluded meta-analysis, confirming the suitability of the chosen scoping review approach. The analysis classified gLLM-driven interventions into 4 key applications, as presented in Table 2. Examination within each category focused on the target tasks assigned to gLLMs, types of input data used, reported dialogue patterns and intervention timing, and performance evaluation methods. The findings of this review demonstrate that existing research has mainly used gLLMs with clinical document inputs for retrospective tasks such as supporting clinical decision-making or extracting data relevant to stroke diagnosis, treatment, prognosis, and rehabilitation. A smaller subset of studies (5/25, 20%) adopted a more patient-centered perspective, either by integrating gLLMs with upper-limb exoskeleton systems to potentially support motor recovery or by applying them to address open-ended patient questions regarding stroke prevention. The single study investigating gLLM use for academic writing support concluded that the outputs were unreliable for practical use, highlighting limitations in that specific application context. Given the breadth of stroke care tasks addressed, considerable diversity in the technical implementation of these gLLM interventions was observed, as shown in Table 3. Common technical approaches involved using GPT-series models, typically accessed through web-based chat interfaces or API calls and guided primarily by task-specific prompt engineering strategies. Moreover, this review identified five critical challenges pertinent to applying gLLMs effectively and safely across the stroke care pathway: (1) ensuring factual alignment, (2) maintaining system robustness, (3) enhancing model interpretability, (4) optimizing operational efficiency, and (5) facilitating adoption into clinical practice. Figure 2 illustrates the current landscape of gLLM-based interventions across the stroke care pathway.

### Need for Rigorous Real-World Evidence to Support Clinical Translation

gLLMs represent a novel addition to digital health [14,61,62], creating new avenues for neurological care [17] and offering significant potential to improve stroke prevention and bridge gaps in care access. Despite this promise, the evidence base for gLLMs specifically in stroke care currently relies heavily on retrospective analyses of clinical documentation and experimental studies conducted in simulated settings. This cautious approach likely reflects valid concerns regarding the potential impact of these nascent technologies on patient safety and clinical decision-making [63]. Highlighting the feasibility of real-world assessment in other domains, a recent cluster-randomized trial in China demonstrated that a gLLM-driven chatbot effectively improved parental health literacy concerning human papillomavirus vaccination for adolescent girls [64]. In contrast, most of the stroke care studies included in this review (24/25, 96%) did not involve integrating gLLM-based systems into actual clinical workflows or conducting real-time interactions with patients. Consequently, the real-world effects of these systems on health care delivery efficiency, clinical outcomes, and patient health literacy within the context of stroke care remain largely unverified. This significant evidence gap highlights an urgent need within the stroke research community. Future efforts must prioritize clarifying evidence requirements and systematically generating robust real-world data on the feasibility, safety, clinical impact, and cost-effectiveness of gLLM applications to provide essential support for clinical translation.

### Toward Balanced Process and Outcome Evaluation

For stroke care tasks that depend on interaction between human users (eg, health care professionals, administrative staff, or patients and their caregivers) and gLLM-based tools, evaluation needs to extend beyond outcome-oriented performance metrics. Incorporating assessments of model reasoning processes and the dynamics of human-gLLM interaction is critical for providing a complete understanding. While 8% (2/25) of the included studies focused solely on noninteractive tasks, including advanced text representation [48,53], the remaining studies (23/25, 92%) relied on human-gLLM interaction to complete stroke care tasks. Among these, more than half (12/23, 52% of the studies) assessed gLLM performance solely based on how well model outputs aligned with clinical expectations or predefined gold standards without assessing human-gLLM interaction processes or model reasoning behavior. While some of these studies (15/25, 60%) aimed to produce correct responses in single-turn dialogues, this narrow, outcome-focused evaluation perspective is insufficient for interventions that rely on gLLMs’ capabilities for open-ended reasoning and interactive engagement [62]. Several studies (11/25, 44%) acknowledged simple process-related metrics in logical coherence, efficiency improvement, and user interaction experience and observed effects. It is also important to examine how well gLLM-driven tools can identify and collect task-relevant information through multiturn interactions, especially in patient-facing contexts [62]. Fully understanding and ensuring the real-world applicability and safety of gLLM-based systems in health care settings requires broadening performance evaluation frameworks to rigorously include these dynamic processes alongside static outcomes.

### Correction of Technical Reporting Deficiencies

Significant issues were raised regarding the normative reporting of gLLM intervention designs within the included studies. A common oversight appeared to be neglecting the fact that different access methods (eg, web-based chat interfaces) may use customized configurations or variants of the same underlying model. This lack of specificity was particularly evident when models were accessed using web chat interfaces. These often used restricted-access [65] or proprietary, fine-tuned, chat-optimized variants [66] (frequently branded as specific products, eg, ChatGPT) that are not directly equivalent to the base models released by developers. Despite researchers’ attempts to specify the underlying models, their precise identity often remained ambiguous. As a result, conflating branded chat products with broader foundation model families (eg, ChatGPT with the GPT series) can lead to conceptual confusion and should be avoided in reporting. Furthermore, this review identified instances in which API-based access to closed-source models was inaccurately characterized, for example, as *static version use* or analogous to offline deployment [50]. In reality, such access depends on remote servers where the underlying models can be updated by the provider without explicit version notification, challenging assumptions of both offline use and version stability. Given the rapid iteration cycles common to gLLMs, consistently time-stamping the input and output stages during use could aid researchers in documenting and interpreting the specific model versions or operational states encountered. However, this practice was uncommon in the reviewed literature, with only 20% (5/25) of the studies reporting time-stamped interaction events [38,40,43,46,57]. To maintain analytical rigor amid these reporting ambiguities, this review adopted a strategy of consistently referring to general model series (eg, the GPT-4 family) when exact versions or configurations could not be definitively ascertained from the studies. The observed heterogeneities and frequent lack of precision in technical reporting highlight a critical need for the development and adoption of standardized, transparent guidelines for describing gLLM-driven intervention designs. Such standards are important for ensuring accurate interpretation, enabling reproducibility, and facilitating meaningful cross-study comparability in this advancing field.

### Simple and Homogeneous Task Adaptation Strategies

The design and refinement of gLLM-driven interventions specifically for stroke care remain in their nascent stages. Current approaches mainly rely on zero- or few-shot instruction designs, enhanced using techniques such as context augmentation, role-based prompting, or format constraints to guide outputs. While prompt iteration was occasionally used to improve factual alignment [42,46,50], generated outputs still often contained inaccuracies or lacked desired nuance. Similarly, although a small subset of the included studies (2/25, 8%) investigated domain-specific fine-tuning of open-source models for better task adaptability, both prompt engineering and basic fine-tuning strategies appear insufficient for highly complex clinical settings that require integrating robust logical reasoning with precise numerical computation. Emerging architectures such as RAG [55,57] and multiagent systems [52] show promise, mirroring developments in other medical fields [67-69], but their empirical validation within stroke care is currently underexplored. Furthermore, the robustness of gLLM-based stroke care interventions against unexpected inputs or variations remains insufficiently examined. The underlying causes of potential failures were often unexplored due to a lack of proactive and systematic investigation strategies within the reviewed studies.

### Underexplored Dual Gap in Human-gLLM Interaction Dynamics

Although intentionally introducing noise or adversarial inputs is a standard method for stress testing and evaluating robustness in machine learning [70], most studies (24/25, 96%) appeared to respond reactively after poor performance was observed, sometimes relying on subjective speculation regarding failure modes rather than rigorous empirical analysis. Systematically analyzing model responses to flawed, edge-case, or adversarial inputs could yield crucial insights into failure mechanisms, thereby informing the development of safer and more reliable gLLMs for stroke care [45,71]. Finally, the rapid iteration cycles and frequent updates of underlying models introduce significant uncertainties regarding the long-term performance, reliability, and transferability of the developed interventions. For example, it remains unclear how effectively interventions initially developed and validated on now deprecated models (eg, early versions of ChatGPT) will function when deployed using substantially updated successor models (such as GPT-4o) [72]. Therefore, this dynamic landscape requires ongoing evaluation, validation, and potentially continuous adaptation strategies for gLLMs intended for clinical use.

While a significant amount of research has focused on gLLM intervention design and technical optimization, how humans interact with such systems within the context of stroke care remains largely underexplored. Although concerns about the *black box* nature of gLLM reasoning processes are frequently discussed, this review suggests that the heterogeneity in users’ subjective interpretations of gLLM outputs presents an equally critical yet less examined challenge. There appears to be emerging agreement on the value of EITL frameworks for deploying gLLMs in real-world settings; however, evidence from the included studies shows that clinicians can interpret the exact same generated response quite differently [46,57]. Such variability in human interpretation may significantly influence downstream trust in the system; subsequent clinical decision-making; and, ultimately, patient outcomes in stroke care.

Beyond interpretation variability, safety concerns are extended by potential user behaviors and governance gaps. For example, follow-up reprompting was reportedly used in one study to bypass built-in safety restrictions designed to prohibit direct radiological image interpretation [40], exposing risks related to both inadequate technology governance and the potential for deliberate misuse by individuals. Moreover, actionable guidelines are urgently needed to address broader safety and ethical concerns, including the legal ambiguities surrounding artificial intelligence–driven interventions and potential conflicts between commercial deployment objectives and established clinical best practices [57].

Consequently, these underexplored dimensions point to a dual gap that limits research and the clinical translation of gLLMs in stroke care. The first gap concerns a limited understanding of optimal gLLM-driven intervention design tailored to specific stroke care tasks, including defining the operational boundaries and failure modes of such systems. The second relates to insufficient investigation into how diverse human users (eg, health care professionals, patients with stroke, and caregivers) actually interact with gLLM-based systems and how these interactions dynamically shape both user understanding and system outputs.

### Future Directions

The application of gLLMs in stroke care, while promising, is relatively new, with most current interventions representing early-stage or relatively simple implementations. To enable the responsible and effective integration of such tools into health care settings, the development and adoption of formal, multidimensional frameworks that promote rigorous evaluation and informed oversight are critical. Future studies attempting to bridge the gap between potential and practice would also likely benefit from using mixed methods techniques to gain deeper, more nuanced insights into how gLLMs actually operate across diverse stroke care tasks and how they can be most effectively and safely deployed in complex clinical environments. In light of the considerations raised in this review, several priorities emerge for guiding the safe, successful, and ethical use of gLLMs across relevant stroke care domains, including clinical work, direct patient support, administrative tasks, and academic research.

First, real-world evidence should be prioritized. There is a critical need for reliable prospective strategies guided by clearly defined research questions and evidence priorities to generate robust real-world data. Such studies should focus on the clinical impact, safety, feasibility, and cost-effectiveness of specific gLLMs implemented in stroke care settings.

Second, transparent technical reporting should be mandated. The technical design and implementation details of gLLM-driven systems must be reported with greater precision and completeness. Standardized reporting should include accurate naming of models or specific product versions used, consistent time-stamping of key input and output events during evaluation, and clear descriptions of how the systems are accessed (eg, through chat interfaces, API, or local deployment).

Third, evaluation frameworks should be broadened beyond output accuracy. Existing performance evaluation for gLLMs requires expansion beyond technical metrics. Future frameworks must incorporate rigorous methods for assessing critical aspects of human-gLLM interaction dynamics, model reasoning processes, context appropriateness, usability, and overall user experience.

Fourth, validation of advanced task adaptation strategies should be strengthened. Current task adaptation strategies in stroke-focused gLLM systems remain simplistic and repetitive, relying primarily on prompt design and inference-time controls. These approaches have shown limitations in handling complex tasks. Future research should develop and evaluate emerging methods (eg, multiagent collaboration and RAG), which are being explored for their feasibility in other areas of chronic disease care.

Finally, mechanisms for safe and effective human-gLLM interaction should be investigated. There is a critical need to clarify the behavioral boundaries and failure modes of gLLM-driven interventions tailored to specific stroke care tasks. Equally important is the lack of empirical insight into how diverse users (eg, health care professionals, patients with stroke, and caregivers) interact with these systems in real-world settings. Future research should elucidate how these interactions shape user understanding and dynamically influence system outputs, supporting the development of more responsive, trustworthy, and context-aware gLLM applications in stroke care.

### Limitations

This review has several limitations related to its scope and the current state of the literature. First, the decision to exclude preprints and focus solely on peer-reviewed publications, while ensuring a certain quality standard, may have omitted important nascent insights given the rapid technological iteration and common use of preprint platforms for early dissemination in the gLLM field. Second, the substantial heterogeneity identified across the included studies precluded a quantitative synthesis or meta-analysis of gLLM intervention performance. To mitigate this, supplementary details summarizing individual study findings are provided (Multimedia Appendix 4 [36-60]) to give readers further granularity where possible. Despite these limitations and the heterogeneity, most reported gLLM-driven interventions demonstrated encouraging performance on their specifically defined tasks within the study contexts. Lower comparative performance was observed in applications focused on extracting structured clinical data, which may reflect the maturity and optimization of existing methods (eg, rule-based systems, conventional machine learning, and earlier deep learning models) already well suited for these specific tasks. In studies targeting knowledge-intensive tasks (eg, lesion detection, report drafting, and evidence integration), mixed or suboptimal results were often reported, likely attributable more to the specific study design used than to an inherent limitation of gLLMs for such tasks generally. Nevertheless, these findings highlight the need for caution regarding the immediate, large-scale deployment or formal adoption of current gLLM-driven interventions in real-world stroke care settings.

### Conclusions

As highlighted throughout this review, current research has yet to establish a coherent, evidence-based foundation addressing robust intervention design, comprehensive multidimensional evaluation, and effective governance for these rapidly evolving gLLM technologies in stroke care. Consequently, this study contributes by clarifying the current complex research landscape concerning gLLM applications in stroke care, providing an updated review of the strengths and critical gaps in existing investigations, and identifying key priorities and directions for future research design and evaluation.

## Appendix

Multimedia Appendix 1 PRISMA-ScR checklist.

Multimedia Appendix 2 Search strategy.

Multimedia Appendix 3 Data extraction variables.

Multimedia Appendix 4 Summary of performance evaluation results for generative large language model–driven interventions in stroke care.

## Abbreviations

API: application programming interface
BART: bidirectional and auto-regressive transformer
EITL: expert-in-the-loop
gLLM: generative large language model
GPT: generative pretrained transformer
NLP: natural language processing
PCC: Population, Concept, and Context
PRISMA-ScR: Preferred Reporting Items for Systematic Reviews and Meta-Analyses extension for Scoping Reviews
RAG: retrieval-augmented generation

## Data Availability

All data generated or analyzed during this study are included in this published article (and Multimedia Appendices 2-4).
