# Supplementary material for: Current Landscape and Future Directions Regarding Generative Large Language Models in Stroke Care: Scoping Review
Source: JMIR Med Inform. 2025 Aug 7;13:e76636. doi: 10.2196/76636 (PMC12371286; doi:10.2196/76636)
Supplement: Multimedia Appendix 2 [file medinform_v13i1e76636_app2.pdf]

Search strategy

Ovid Embase

Run 24/12/2024

ID	Term	Hits
1	stroke.mp.	614248
2	apoplex*.mp.	4734
3	1 or 2	618303
4	("pretrained language model" or "large language model" or "generative pretrained transformer" or GPT* or "multi-agent" of "natural language processing" or avatar or chatbot).mp.	38706
5	(generat* adj2 (model or "artificial intelligence")).mp.	20261
6	exp generative pretrained transformer/	4736
7	exp generative artificial intelligence/	5839
8	exp generative model/	5370
9	exp large language model/	6500
10	exp ChatGPT/	4464
11	exp avatar/	283
12	exp chatbot/	5677
13	exp artificial intelligence chatbot/	4724
14	exp multi-agent system/	595
15	4 or 5 or 6 or 7 or 8 or 9 or 10 or 11 or 12 or 13 or 14	59932
16	(("virtual" or "digital") adj3 ("assistant" or "coach" or "caregiver" or "patient" or "nurs*" or "physician" or "peer" or "partner" or "mate" or "tutor" or "advisor" or "consultant" or "specialist" or "teacher" or "therapist")).mp.	10655
17	exp virtual assistant/	503
18	16 or 17	10655
19	(("convers*" or "dialog*" or "text based" or "educat*" or "chat*") adj3 ("agent" or "assistant" or "robot")).mp.	1593
20	14 or 17 or 18	71475
21	3 and 19	911

PubMed

Run 24/12/2024

ID	Term	Hits
1	(Stroke[MeSH Terms]) OR (stroke[Title/Abstract]) OR (apoplex*[Title/Abstract])	387168
2	("pretrained language model" [Title/Abstract]) OR ("large language	28132

	model"[Title/Abstract]) OR ("generative pretrained transformer" [Title/Abstract]) OR ("GPT"[Title/Abstract]) OR (“generat* model”[Title/Abstract]) OR ("generat* artificial intelligence"[Title/Abstract]) OR ("multi-agent"[Title/Abstract]) OR ("avatar"[Title/Abstract]) OR ("chatbot"[Title/Abstract]) OR ("natural language processing"[Title/Abstract])	
3	Avatar [MeSH Terms]	68
4	("virtual"[Title/Abstract] OR "digital"[Title/Abstract]) AND ("assistant"[Title/Abstract] OR "coach"[Title/Abstract] OR "caregiver"[Title/Abstract] OR "patient"[Title/Abstract] OR "nurs*"[Title/Abstract] OR "physician"[Title/Abstract] OR "peer"[Title/Abstract] OR "partner"[Title/Abstract] OR "mate"[Title/Abstract] OR "tutor"[Title/Abstract] OR "advisor"[Title/Abstract] OR "consultant"[Title/Abstract] OR "specialist"[Title/Abstract] OR "teacher"[Title/Abstract] OR "therapist" [Title/Abstract])	65786
5	("convers*"[Title/Abstract] OR "dialog*"[Title/Abstract] OR "text based"[Title/Abstract] OR "educat*"[Title/Abstract] OR "chat*"[Title/Abstract]) AND ("agent"[Title/Abstract] OR "assistant"[Title/Abstract] OR "robot"[Title/Abstract])	20490
6	#2 OR #3 OR #4 OR #5	112627
7	#1 AND #6	1988

Scopus
Run: 24/12/2024

ID	Term	Hits
1	TITLE-ABS-KEY(stroke OR apoplex*)	583394
2	TITLE-ABS-KEY ({pretrained language model} OR {large language model} OR { generative pretrained transformer} OR "GPT" OR {natural language processing} OR "multi-agent" OR OR "avatar" OR "chatbot")	293545
3	TITLE-ABS-KEY ((generat*) W/2 (model OR “artificial intelligence”))	191873
4	TITLE-ABS-KEY (("virtual" OR "digital") W/3 ("assistant" OR "coach" OR "caregiver" OR "patient" OR "nurs*" OR "physician" OR "peer" OR "partner" OR "mate" OR "tutor" OR "advisor" OR "consultant" OR "specialist" OR "teacher" OR "therapist"))	43185
5	TITLE-ABS-KEY (("convers*" OR "dialog*" OR "text based" OR	24114

	"educat*" OR "chat*") W/3 ("agent" OR "assistant" OR "robot"))	
6	#1 AND (#2 OR #3 OR #4)	2318

CINAHL Plus with Full Text

Run 24/12/2024

Result:

ID	Term	Hits
1	AB (stroke OR apoplex*) OR TI (stroke OR apoplex*)	116656
2	AB ("pretrained language model" OR "large language model" OR "generative pretrained transformer " OR "GPT" OR "natural language processing" OR "multi-agent" OR "avatar" OR "chatbot") OR TI ("pretrained language model" OR "large language model" OR "generative pretrained transformer " OR "GPT" OR "natural language processing" OR "multi-agent" OR "avatar" OR "chatbot")	4188
3	AB ("generat*" AND ("model" OR "artificial intelligence")) OR TI("generat*" AND ("model" OR "artificial intelligence"))	22402
4	AB (("virtual" OR "digital") AND ("assistant" OR "coach" OR "caregiver" OR "patient" OR "nurs*" OR "physician" OR "peer" OR "partner" OR "mate" OR "tutor" OR "advisor" OR "consultant" OR "specialist" OR "teacher" OR "therapist")) OR TI (("virtual" OR "digital") AND ("assistant" OR "coach" OR "caregiver" OR "patient" OR "nurs*" OR "physician" OR "peer" OR "partner" OR "mate" OR "tutor" OR "advisor" OR "consultant" OR "specialist" OR "teacher" OR "therapist"))	20856
5	AB (("convers*" OR "dialog*" OR "text based" OR "educat*" OR "chat*") AND ("agent" OR "assistant" OR "robot")) OR TI (("convers*" OR "dialog*" OR "text based" OR "educat*" OR "chat*") AND ("agent" OR "assistant" OR "robot"))	4512
6	S2 OR S3 OR S4 OR S5	50998
7	S1 AND S6	933

Web of Science Core Collection

Run:24/12/2024

ID	Term	Hits
1	TS=(stroke OR apoplex*) and Preprint Citation Index (Exclude – Database)	1031762
2	TS=("pretrained language model" OR "large language model" OR "generative pretrained transformer" OR "GPT" OR "multi-	322027

	agent"OR "natural language processing" OR "avatar" OR "chatbot") and Preprint Citation Index (Exclude – Database)	
3	TS=((generat*) NEAR/2 (model OR "artificial intelligence")) and Preprint Citation Index (Exclude – Database)	318900
4	TS=(("virtual" OR "digital") NEAR/3 ("assistant" OR "coach" OR "caregiver" OR "patient" OR "nurs*" OR "physician" OR "peer" OR "partner" OR "mate" OR "tutor" OR "advisor" OR "consultant" OR "specialist" OR "teacher" OR "therapist")) and Preprint Citation Index (Exclude – Database)	332564
5	TS=(("convers*" OR "dialog*" OR "text based" OR "educat*" OR "chat*") NEAR/3 ("agent" OR "assistant" OR "robot")) and Preprint Citation Index (Exclude – Database)	19060
6	#2 OR #3 OR #4 OR #5 and Preprint Citation Index (Exclude – Database)	975133
7	#6 AND #1 and Preprint Citation Index (Exclude – Database)	3695
8	#6 AND #1 and Preprint Citation Index (Exclude – Database) and Patent or Retracted Publication or Awarded Grant (Exclude – Document Types)	2244

IEEE Xplore

Run:24/12/2024

ID	Term	Hits
1	(("All Metadata": stroke) OR ("All Metadata": apoplex*)) AND ((("All Metadata": "pretrained language model") OR ("All Metadata": "large language model") OR ("All Metadata": "generative pretrained transformer") OR ("All Metadata": "GPT") OR ("All Metadata": "natural language processing") OR ("All Metadata": "multi-agent") OR ("All Metadata": "avatar") OR ("All Metadata": "chatbot")) OR (("All Metadata": generat*) NEAR/2 (("All Metadata": model) OR ("All Metadata": "artificial intelligence"))) OR ((("All Metadata": "virtual") OR ("All Metadata": "digital")) NEAR/3 (("All Metadata": "assistant") OR ("All Metadata": "coach") OR ("All Metadata": "caregiver") OR ("All Metadata": "patient") OR ("All Metadata": "nurs*") OR ("All Metadata": "physician") OR ("All Metadata": "peer") OR ("All Metadata": "partner") OR ("All Metadata": "mate") OR ("All Metadata": "tutor") OR ("All Metadata": "advisor") OR ("All Metadata": "consultant") OR ("All Metadata": "specialist") OR ("All	391

	Metadata": "teacher") OR ("All Metadata": "therapist"))) OR ((("All Metadata": "convers*") OR ("All Metadata": "dialog*") OR ("All Metadata": "text based") OR ("All Metadata": "educat*") OR ("All Metadata": "chat*")) NEAR/3 (("All Metadata": "agent") OR ("All Metadata": "assistant") OR ("All Metadata": "robot"))))	
