# Supplementary material for: Current Landscape and Future Directions Regarding Generative Large Language Models in Stroke Care: Scoping Review
Source: JMIR Med Inform. 2025 Aug 7;13:e76636. doi: 10.2196/76636 (PMC12371286; doi:10.2196/76636)
Supplement: Multimedia Appendix 3 [file medinform_v13i1e76636_app3.pdf]

Data extraction variables

1. Citation (include the title, publication type, the first author's last name, and the year of publication)
2. Country of the study (the country where the research occurs, along with the national backgrounds of the involved medical staff and patients)
3. Care stage (include prevention, diagnosis, treatment, prognosis, or rehabilitation; if more than two stages, list them all)
4. Study design (if there are multiple design features, record the most relevant and accurate one)
5. Characteristics of stroke patient populations (include sample size; if specific numbers are unavailable, record the related data scale as accurately as possible; percentage of males; age mean or median, along with standard deviation or interquartile range); stroke phenotype; comorbidities; stroke outcome scoring tool: score mean or median, along with standard deviation or interquartile range)
6. Generative large language model (gLLM)-driven intervention design (foundation model or model series; model access, including application programming interface, web-based chat interface, local inference, or others; instruction design, including zero-shot, few-shot, context-enhanced, role-based, or format-constrained; model adaptation, with a focus on strategies for hyperparameters and model architecture changes)
7. Objective tasks of gLLM-driven interventions
8. Input data or sources
9. Dialogue patterns (include single-turn, multi-turn, or not applicable; if more than two types, list them all)
10. Timestamp of reported input or output events
11. Gold-standard providers or benchmarks
12. Performance evaluation perspectives
13. Performance evaluation metrics
14. Key results of the intervention implementation
