# Supplementary material for: Current Landscape and Future Directions Regarding Generative Large Language Models in Stroke Care: Scoping Review
Source: JMIR Med Inform. 2025 Aug 7;13:e76636. doi: 10.2196/76636 (PMC12371286; doi:10.2196/76636)
Supplement: Multimedia Appendix 4 [file medinform_v13i1e76636_app4.pdf]

Summary of performance evaluation results for generative large language model–driven interventions in stroke care

Study	Task objectives	Foundation model or model series	Access	Performance evaluation results
Clinical decision-making support (n=10)				
Pedro et al [38]	Predict the mRS[a] score at 3 months after mechanical thrombectomy	GPT-3.5	Web-based chat interface (ChatGPT)	A κ values of 0.354 for exact agreement and 0.727 for dichotomized agreement were achieved with the 3-month mRS, outperforming the MT-DRAGON[b].
Chen et al [39]	Make clinical decisions for mechanical thrombectomy	GPT-4	Web-based chat interface (ChatGPT)	A match rate of 54.3% with clinician decisions was observed. Mechanical thrombectomy decisions were conservative and aligned with clinical guidelines, with errors primarily related to mathematical reasoning, followed by logical inconsistencies and data misinterpretation.
Strotzer et al [40]	Interpret MRI[c] and CT[d] images and generate free-text reports in stroke cases	GPT-4- 1106-vision-preview	Official API[e] (via OpenAI platform)	Accurate generation of imaging modality and anatomic region was achieved in free-text reports, with consistent output across runs for both elements. Ischemic stroke was identified with 46% accuracy, 56% sensitivity, and 36% specificity; hemorrhage was detected with 54% accuracy, 100% sensitivity, and 8% specificity. Only limited benefit was observed for the nonradiologist in training.
Kuzan et al [41]	Interpret DWI[f] and ADC[g] maps in	GPT-4 Vision	Web-based chat	MRI sequence, diffusion restriction, and the affected

	acute stroke cases		interface (ChatGPT)	brain region were correctly identified with accuracies of 88.3%, 79.5%, and 26.2%, respectively. Stroke was diagnosed with 79.6% sensitivity, 84.9% specificity, a positive predictive value of 83.9%, and a negative predictive value of 80.8%.
Fei et al [42]	Evaluate cognitive performance in stroke cases	GPT-3.5 and GPT-4	Web-based chat interface (ChatGPT)	Compared to the GPT-3.5 model series, the GPT-4 model series showed greater reliability and closer alignment with physician judgments.
Lee et al [43]	Locate lesions based on patient H&P[h] text	GPT-4	Unclarified	Strong and consistent performance was observed across sides and brain regions, with the exception of the cerebellum. Errors were primarily attributed to limited case information, followed by insufficient pretrained knowledge and logical failure.
Haim et al [44]	Calculate the NIHSS[i] score and predict the use of tissue plasminogen activator	GPT-3.5 and GPT-4	Web-based chat interface (ChatGPT)	The GPT-4 model series demonstrated better agreement with two physicians (κ=0.473 and 0.576) and a higher AUC-ROC[j] (0.644), compared to the GPT-3.5 model series (κ=0.289 and 0.302; AUC-ROC=0.630).
Chen et al [45]	Calculate GCS[k], H&H[l], and ICH[m] scores	GPT-4	Web-based chat interface (Bing chat)	The average error rate and average error magnitude were 10% and 0.15 for the GCS, 13% and 0.13 for the H&H, and 27.5% and 0.325 for the ICH scoring, with higher average error rate under conditions of missing data and simplified

				prompts.
Blacker et al [46]	Use SNACC[n] HQRs[o] to answer questions on perioperative stroke and endovascular treatment anesthesia	GPT-4	Web-based chat interface (ChatGPT)	The responses fail to include over 50% of HQRs, vary in clinical interpretation, lack reliable references, and may contain harmful content.
Zhang et al [37]	Generate rehabilitation prescriptions and ICF[p] codes in a stroke case	GPT-4	Web-based chat interface (ChatGPT)	Rehabilitation prescriptions and corresponding ICF codes are accurately generated with appropriate rationale, though responses remain overly general and include an ICF category misclassification.
Administrative assistance (n=9)				
Sivarajkumar et al [47]	Extract and categorize physical rehabilitation exercise information from stroke cases	GPT-3.5-turbo	Official API (via Microsoft Azure)	Recall is notable, but precision and F_1-score are lower than those achieved by rule-based methods and machine learning algorithms.
Guo et al [48]	Extract triples by fine-tuning and integrating a relation classification module	BART[q]-base-Chinese and BART-large-Chinese	Unclarified	F_1-score improvements ranged from 0.37% to 1.64% for JREwBART[r] and from 0.26% to 2.00% for PRE-BARTaBT[s], compared to the baseline model, Cas-CLN[t].
Lehnen et al [49]	Extract key information for mechanical thrombectomy	GPT-3.5 and GPT-4	Web-based chat interface (ChatGPT)	An exact match rate of 93.16% was achieved by the GPT-4 model series and 63.93% by the GPT-3.5 model series, with errors in content and format identified, and the GPT-4 model series consistently outperforming the GPT-3.5 model series.

Fiedler et al [50]	Extract IPSS[u] format information and infer disease severity	GPT-3.5-turbo-16k	Official API (via Microsoft Azure)	Following iterative prompt refinement, an exact match rate of 93.16% was achieved.
Wang et al [51]	Extract and infer key information for mechanical thrombectomy	GPT-3.5-turbo, GPT-4, Gemini Pro, GLM-4, Spark 3, and Qwen-Max	Official APIs (via unclarified platforms)	Item-level accuracy reached 95.09% and overall accuracy 78.05% across six model series, with an average processing time of 73.10 seconds per case. GLM-4 and the GPT-4 model series demonstrated leading performance in advanced extraction tasks.
Goh et al [52]	Extract stroke audit data	Llama 3-70B	Local inference	An exact match rate of 93.8% was achieved, with correctness confirmed in 29 of 38 clinician-disputed entries (76.3%).
Baro et al [53]	Predict stroke hospitalization by fine-tuning and integrating classification layers	openCabrita 3B	Unclarified	The highest sensitivity of 93.5% was observed for total prediction, along with the highest F_1-score and AUC[v] across prediction windows. Stroke-specific fine-tuning yielded a specificity of 87% and outperformed general models.
Meddeb et al [54]	Extract key information for mechanical thrombectomy items	Qwen-72B, Mixtral 8x7B, and BioMistral-7B	Local inference	The best performance was observed for the Mixtral model series, followed by the Qwen and BioMistral model series. An average time savings of 65.6% per case was achieved with the expert-in-the-loop approach.
Kim et al [55]	Perform data wrangling on a large dataset of patients with stroke	GPT-4-32k	Official API (via unclarified platform)	Improve clinicians' ability to reliably and efficiently obtain meaningful clinical insights from large-scale and complex clinical datasets.
Direct patient interaction (n=5)				

Argymbay et al [56]	Provide personalized stroke risk insights and answer medical queries based on patient data	BioMistral-7B	Private API (via Hugging Face on Amazon SageMaker)	Provide a detailed review of risk factors, along with personalized recommendations for lifestyle modifications and medical guidance to address patient concerns.
Neo et al [57]	Answer rehabilitation questions for patients with stroke and their caregivers	GPT-3.5-turbo and PaLM 2	Web-based chat interfaces (ChatGPT and Google Bard)	Similar overall performance was observed between the two models, with the PaLM 2 model series slightly outperforming the GPT-4 model series in terms of relevance and safety. Inter-rater agreement was fair at best.
Wu et al [58]	Provide nonmedical professionals with stroke-related health information	GPT-3.5	Web-based chat interface (ChatGPT)	ChatGPT's responses required a lower reading grade level than those of Google Assistant. However, the reading levels for both systems remained above the high school level.
Chen et al [59]	Interpret commands and generate Python code for hand exoskeleton control	GPT-4, GPT-3.5-turbo, and GLM-130B	Official APIs (via unclarified platforms)	Three model series demonstrated potential for exoskeleton control. The GPT-4 model series achieved higher task success rates than the GPT-3.5 model series, while the GLM-130B model series showed the lowest performance. Execution time was slightly longer for GPT-4 compared to GPT-3.5, with GLM-130B requiring the longest time.
Rifai et al [60]	Interpret commands and generate target coordinates for upper-limb robot control	GPT-4o	Official API (via unclarified platforms)	Performance exceeded that of the traditional joystick-based interface.
Automated literature review (n=1)				

Anghelescu et al [36]	Assist in obtaining evidence on Actovegin's efficacy for ischemic stroke	Unclarified GPT[w]	Web-based chat interface (ChatGPT)	A broad overview of current scientific knowledge was provided. However, instances of fabricated academic content were identified, and the system was unable to produce evidence syntheses aligned with the PRISMA guidelines.

[a]mRS: modified Rankin Scale.

[b]MT-DRAGON: mechanical thrombectomy-DRAGON score.

[c]MRI: magnetic resonance imaging.

[d]CT: computed tomography.

[e]API: application programming interface.

[f]DWI: diffusion-weighted imaging.

[g]ADC: apparent diffusion coefficient.

[h]H&P: history and neurological physical examination.

[i]NIHSS: National Institutes of Health Stroke Scale.

[j]AUC-ROC: area under the receiver operating characteristic curve.

[k]GCS: Glasgow Coma Scale.

[l]H&H: Hunt and Hess scale.

[m]ICH: intracranial hemorrhage.

[n]SNACC: Society for Neuroscience in Anesthesiology and Critical Care.

[o]HQR: high-quality recommendation.

[p]ICF: International Classification of Functioning, Disability, and Health.

[q]BART: bidirectional and auto-regressive transformers.

[r]JREwBART: joint entity relation extraction with BART.

[s]PRE-BARTaBT: pipeline entity relation extraction based on the BART and biaffine transformation

[t]Cas-CLN: the cascade binary pointer tagging network with conditional layer normalization.

[u]IPSS: International Pediatric Stroke Study.

[v]AUC: area under the curve.

[w]GPT: generative pretrained transformer.
